# Is synesthesia more common in patients with Asperger syndrome?

**DOI:** 10.3389/fnhum.2013.00847

**Published:** 2013-12-09

**Authors:** Janina Neufeld, Mandy Roy, Antonia Zapf, Christopher Sinke, Hinderk M. Emrich, Vanessa Prox-Vagedes, Wolfgang Dillo, Markus Zedler

**Affiliations:** ^1^Centre for Integrative Neuroscience and Neurodynamics, School of Psychology and Clinical Language Sciences, University of ReadingReading, UK; ^2^Department of Clinical Psychiatry, Social Psychiatry, and Psychotherapy, Hannover Medical SchoolHannover, Germany; ^3^Deparment of Medical Statistics, Georg-August-UniversityGoettingen, Germany; ^4^Department of Neurology, University Medical Center Hamburg-EppendorfHamburg, Germany

**Keywords:** Asperger syndrome, autism, synesthesia, prevalence, development

## Abstract

There is increasing evidence from case reports that synesthesia is more common in individuals with autism spectrum conditions (ASC). Further, genes related to synesthesia have also been found to be linked to ASC and, similar to synaesthetes, individuals with ASC show altered brain connectivity and unusual brain activation during sensory processing. However, up to now a systematic investigation of whether synesthesia is more common in ASC patients is missing. The aim of the current pilot study was to test this hypothesis by investigating a group of patients diagnosed with Asperger Syndrome (AS) using questionnaires and standard consistency tests in order to classify them as grapheme-color synaesthetes. The results indicate that there are indeed many more grapheme-color synaesthetes among AS patients. This finding is discussed in relation to different theories regarding the development of synesthesia as well as altered sensory processing in autism.

## Introduction

### Synesthesia

Synesthesia is a non-pathological phenomenon in which specific sensory stimuli (e.g., a sound) or concepts (e.g., time units or numbers) lead automatically to additional, internally generated sensations (e.g., colors, textures or shapes). A stimulus leading to synaesthetic sensations is termed “inducer,” while the internally generated synaesthetic sensation is termed ‘concurrent’ (Grossenbacher and Lovelace, 2001). The most investigated type of synesthesia is grapheme-color synesthesia (GCS) in which numbers and/or letters are perceived in specific colors, occurring in about 1.1–2.0% of the population (Simner et al., 2006). The consistency of the specific synaesthetic couplings, i.e., that a certain letter always triggers a sensation of the same color, has been defined as one of the key features of synesthesia (Cytowic, 1995) and it has been shown to persist over long time periods (Simner and Logie, 2007). Although the view that synaesthetic coupling is always consistent over time has been questioned recently (Simner, 2012), consistency tests (Baron-Cohen et al., 1987; Eagleman et al., 2007) are still used as gold standard to distinguish synaesthetes from non-synaesthetes. A standardized online test battery (http://www.synesthete.org) has been developed for the classification of GCS, as well as other types of synesthesia (e.g., auditory-visual synesthesia, spatial-sequence synesthesia, etc.). However, up to now the test for GCS is the most commonly used and its potential to differentiate synaesthetes from non-synaesthetes has been validated (Eagleman et al., 2007). Another key characteristic of synesthesia is the automaticity of consistent couplings, which can be tested with a speeded consistency test where inducers are presented either in a synaesthetically congruent or incongruent color (Eagleman et al., 2007). The idea of this test is that, as synesthesia occurs automatically, synaesthetes should be able to quickly distinguish synaesthetically congruent from incongruent trials. Indeed, synaesthetes have been shown to respond much more accurately in this test than controls.

As synesthesia runs in families, it is likely to have a genetic component: about 40–50% of synaesthetes report a first-degree relative who is also a synaesthete (Baron-Cohen et al., 1996; Barnett et al., 2008a,b). Further, specific gene loci have been identified as being related to this phenomenon (Asher et al., 2009; Tomson et al., 2011). However, as different types of synesthesia can occur within the same family, and as the synaesthetic experiences are highly individual for each synaesthete, it has been suggested that a genetic predisposition to develop synesthesia exists in general, but that the development of individual couplings is influenced by environmental factors (Barnett et al., 2008a,b).

The neuronal mechanisms underlying synesthesia still remain to be clarified. In GCS, there is evidence of the involvement of visual, parietal, and frontal brain areas (Rouw et al., 2011), whereas involvement of the parietal cortex has also been found in sequence-space (Tang et al., 2008) and (language-unrelated) auditory-visual synesthesia (Neufeld et al., 2012a).

### Indications for a relationship between autism spectrum conditions (ASC) and synesthesia

Together with his co-workers, Simon Baron-Cohen published a case study on a remarkable man with synesthesia, Asperger Syndrome (AS) and savantism (Bor et al., 2007). They suggested that co-occurrence of AS and synesthesia might increase the likelihood of savantism. But there is also evidence for a linkage between ASC and synesthesia. While synesthesia is characterized by additional, internally generated sensations and in relation to that also often by unusual brain activation in sensory cortex regions (Rouw et al., 2011), altered sensory processing and sensory symptoms are also commonly found in individuals with ASC, e.g., in the visual (Simmons et al., 2009) and auditory domain (O'Connor, 2012; Samson et al., 2006). According to a recent review, sensory hypo- and hyper responsiveness are more frequent in ASC than in other developmental disabilities and atypical neural activity following sensory stimulation has been found in ASC patients even at the level of the primary sensory cortex in the auditory, tactile, and visual domain (Marco et al., 2011). It has been demonstrated that auditory stimuli can trigger responses in both auditory and nearby visual brain regions in autistic individuals (Kemner et al., 1995). Similarly, co-activation of the color processing area in the fusiform gyrus has been found in GCS (Hubbard, 2007) in response to the presentation of colorless graphemes. Further, increased connectivity has been detected in the brains of individuals with autism (Courchesne et al., 2005) as well as in synaesthetes (Rouw and Scholte, 2007; Jaencke et al., 2009; Haenggi et al., 2011). Findings showing altered brain anatomy in autistic individuals have led to the hypothesis that there is a developmental bias in ASC toward forming more short range connections, leading to hyper connectivity of local networks (Casanova and Trippe, 2009). Moreover, it has been suggested that cross-activation of adjacent brain areas are the mechanism underlying synesthesia (Hubbard and Ramachandran, 2005).

Besides these similarities, there is some evidence for a genetic linkage between ASC and synesthesia. Cytowic pointed out that about 15% of synaesthetes report having a first-degree relative with dyslexia, autism or attention deficit disorder (ADD) (Cytowic, 1995). A recent whole genome investigation with auditory-visual synaesthetes revealed a significant linkage of this type of synesthesia to genes which have previously been shown to be linked to autism (Asher et al., 2009; IMGSAC, 2001).

Finally, there is some anecdotal evidence for synesthesia in ASC patients (Harrison and Hare, 2004) and preliminary data based on self-report suggest that synesthesia is more common in individuals with AS (Johnson et al., 2011).

However, a systematic investigation into the relationship between AS and synesthesia using standardized tests is missing up to now. Here we tested a group of individuals diagnosed with ASC for GCS. We hypothesized that this type of synesthesia is more common in individuals with ASC.

## Methods

### Diagnostic process

DSM-IV criteria for AS in child- and adulthood (A.P.A., 1994) were thoroughly explored by a self-developed semi-structured interview (“Diagnostic interview: AS in adulthood”). After a general medical anamnesis (somatic, psychiatric and social history, including childhood development) the second part specifically explored symptoms related to AS. This part contained the sections: social interaction and communication (e.g., empathic abilities, friendships and interest in peers); special interests (e.g., in specific objects/topics); stereotype behavior (e.g., rituals, reaction to disturbance of rituals); and other characteristics (e.g., clumsiness, increased sensitivity toward sensory stimuli). Each section addressed child- and adulthood separately. Additionally, eye contact, facial expressions, prosody, “mirroring” of affections, and clumsiness were observed during the interview. The duration of the interview was about 90 min. Diagnosis was completed with information from personal interviews, either by telephone or in written form, with observers in child- and/or adulthood, such as partners, friends, parents or siblings. In some cases, school reports completed anamnesis.

All patients were interviewed by the same experienced investigator. Diagnosis was only confirmed if all DSM-IV criteria were clearly fulfilled. Retrospective data on the development of speech were assessed. Two male patients were excluded from the study retrospectively, as the possibility of delayed speech onset could not be reliably excluded in these cases.

Additionally we used two self-rating scales to complement diagnosis: the autism-spectrum quotient (AQ) (Baron-Cohen et al., 2001) and the empathy quotient (EQ) (Baron-Cohen and Wheelwright, 2004). The AQ is an instrument for quantifying where an individual is placed on the continuum from typical to autistic, a high score indicating more pronounced autistic traits. The AQ consists of 50 items which are divided into five subscales: social skill, attention switching, attention to detail, communication, and imagination. The EQ is an instrument for estimating the empathic abilities of an individual and a high score indicates greater empathy. It consists of 40 items on empathy and 20 filler items. Baron-Cohen suggested a cut-off score of ≥32 points for the AQ (80% of patients with AS scored more than 32 points) and a cut-off score of ≤30 points for the EQ (81% of patients with AS scored less than 30 points). However, the score for 20% of Baron-Cohen's patients was outside these cut-offs. Therefore those patients whose score was outside the autistic range were also included, as long as they fulfilled the DSM-IV criteria for AS.

Every patient was examined for axis-I co-morbidity by using the German version of the Structured Clinical Interview for DSM-IV Axis I Disorders (SCID-I) (Wittchen et al., 1997).

### Participants

All patients diagnosed with AS between February 2008 and June 2011 in the Clinic for Psychiatry of the Hannover Medical School (*n* = 29, 8 women) received a written or oral invitation to participate in the study, for which a small amount of monetary compensation was offered. The study was described as involving computer tests and questionnaires. Nothing was mentioned about synesthesia before participation to avoid a recruiting bias. In all, 21 patients (5 women, mean age = 37.9 ± 11.3 years) gave informed consent to participate in the study and completed the tests, as well as the questionnaires. The study was approved by the local ethics committee.

### Experimental procedure

**Figure 1:**
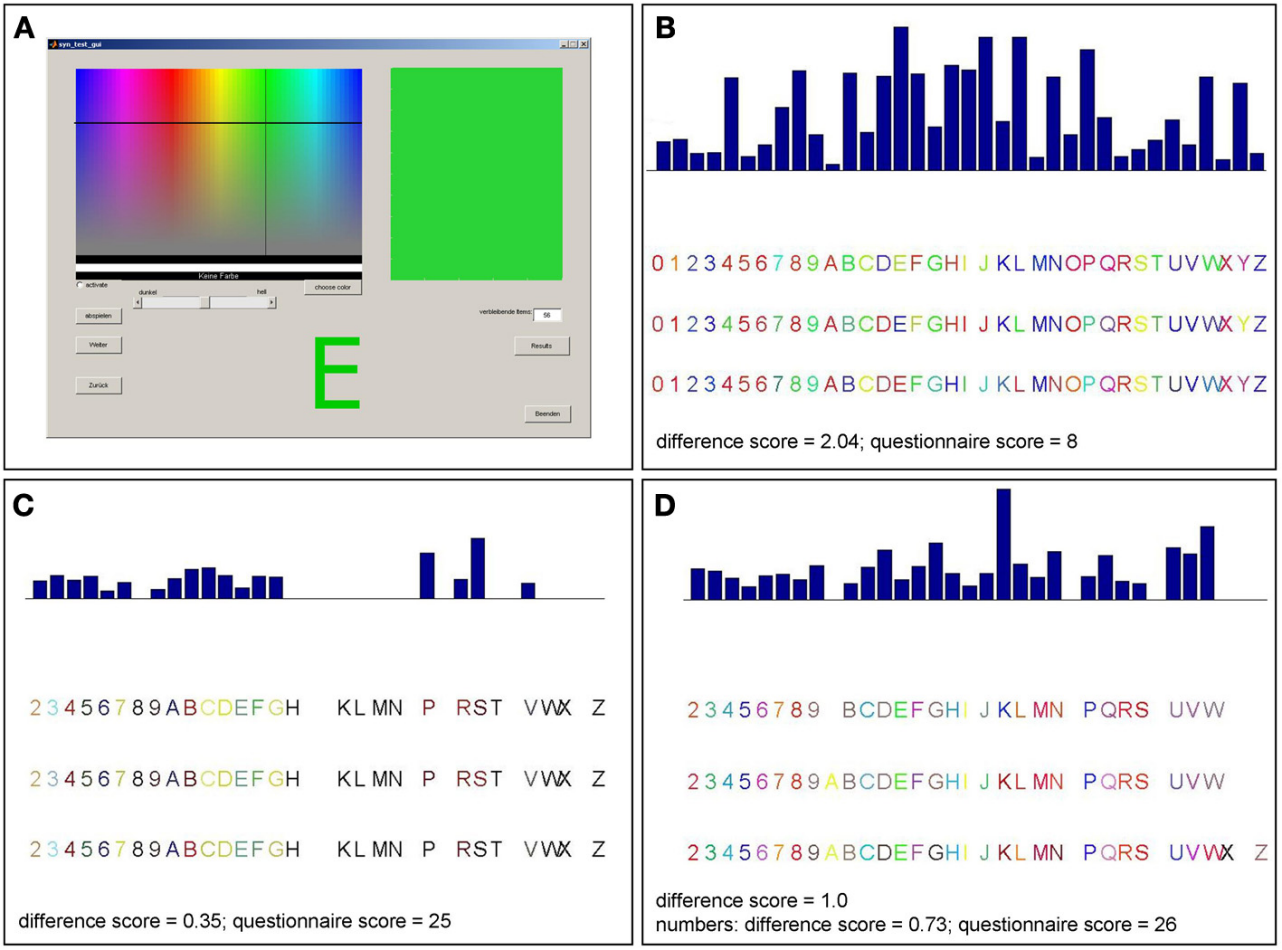
**Graphical surface of the consistency test and examples of consistency test results for different patients. (A)** Participants selected a color for each item presented on the screen by moving a cross-hair cursor over a color matrix. The majority of patients reported not perceiving colors when seeing numbers or letters and made inconsistent color choices as in **(B)**. Accordingly, those patients have rather high scores in the consistency test (>1.0). This is consistent with their reports and their low scores in the six-point questionnaire (<17). Three patients made consistent color choices (score <1.0) for letters and numbers **(C)** or numbers only **(D)** which was consistent with their reports and six-point questionnaire scores (those patients claiming to perceive synesthesia differently for numbers and letters completed the questionnaire twice, separately for numbers and letters).

Once a participant arrived, he received a short description of synesthesia. A few examples of the different types of synesthesia, including GCS, were described. Furthermore, it was explained that synesthesia does not mean associations (e.g., associating the word “love” with the color red, or metaphorical associations, such as “being angry” = “seeing red”). Most of the participants claimed that they never heard of synesthesia before. After the introduction, participants were asked whether they experience color sensations when seeing numbers or letters. Participants then performed a consistency test for GCS (Eagleman et al., 2007), an offline version of the Synesthesia Test Battery (http://www.synesthete.org) on a PC. During this test, the numbers from 0 to 9 and the 26 letters of the alphabet were presented in black ink on white background on a computer screen. Each stimulus was presented separately and three times in a randomized order, so that there were 108 stimuli in total. Subjects were instructed to choose the color which they thought best fitted the presented grapheme by moving a cross-hair cursor on to a color matrix (see Figure 1A). As there was no time pressure to choose the colors in this test, the completion time varied (from about 20 min to 1 h).

After the test, participants completed a short questionnaire, a German version of the questionnaire used in the prevalence investigation by Simner et al., consisting of six questions (see supplementary material) which were rated on a six-point Likert scale to assess each participant's subjective view concerning consistency and vividness of potentially synaesthetic sensations experienced during the consistency test (Simner et al., 2006). Participants were asked whether their answers to these questions would be the same for numbers and letters and, if that was not the case, to fill in the questionnaire twice, once for numbers and once for letters. Responses ranged from “strongly disagree” (“trifft gar nicht zu”) to “strongly agree” (“trifft voll zu”) and were coded from 0 to 5. Scores between 0 and 30 could be achieved, with higher numbers indicating synesthesia (the highest score per question, 5, was assigned to “strongly agree” for questions 1, 3, 5, and 6 and to “strongly disagree” for questions 2 and 4). As a group of 20 known grapheme-color synaesthetes scored on average 26.4 in this questionnaire (*SD* = 4.64), the synaesthetic range has been defined as a score between 17 and 30 (lower bound = two standard deviations below mean score) (Simner et al., 2006).

In order to prevent false classification of participants using memorizing strategies to give consistent responses like synaesthetes, the consistency test was followed by a speeded consistency test. The latter is designed to test for the criterion of automaticity and is based on the assumption that the possibility of giving correct responses from memory is ruled out if the response has to be given quickly. (Eagleman et al., 2007). In this task, participants see a colored grapheme presented on the screen for 1 s. In 50% of trials, the color is congruent with the synaesthetic color reported by the participant, in the other 50% of trials the color is incongruent, thus different from that color. In each trial, participants are instructed to report as quickly as possible by pressing a button whether the color of the letter is congruent with their synaesthetic color or not. The test consists of 108 trials and takes approximately 10 min.

Participants were only classified as synaesthetes if they fulfilled all three criteria: (1) subjective experience of synesthesia (assessed by self-report and short questionnaire), (2) consistency (indicated by a consistency score lower than 1.0 for those items for which synesthesia was reported) and (3) automaticity of synaesthetic sensations (indicated by an accuracy level of at least 80% in the speeded consistency test).

At the end, an extensive interview, based on a synesthesia questionnaire, was conducted to obtain additional information about the subjective experience of GCS, as well as other synesthesia types, and to collect some data on the patients (age, sex, education, etc.). One part of the synesthesia questionnaire was a list of possible possible inducers and concurrents, as in the questionnaire by Simner et al. (2006). Participants were asked to indicate any of synesthesia they thought they may have by drawing lines on the questionnaire between listed inducers and concurrents. They were further asked to name any additional type of synesthesia involving inducers or concurrents which were not on the list, or to modify items to describe the sensation in a better way. All in all, the whole investigation took approximately 2 h per subject.

### Data analysis

#### Consistency test

As GCS is characterized by stable grapheme-color mapping, color consistency was calculated for the color choices per grapheme made during the three runs. The color variation was calculated from the geometric distance of the RGB (red green blue) values chosen for each item within the three trials. A consistency score was then calculated as the mean geometric distance per item over all N items (Eagleman et al., 2007). N was 36 – X (*X* = the number of items for which the “no color” button was chosen). Synaesthetes usually achieve scores smaller than 1.0, indicating greater consistency, while controls usually achieve higher scores (on average 2.0) indicating less consistent color choices.

#### Speeded consistency test

The percentage of correct responses in all 108 trials, as well as the mean response time, was then calculated. Synaesthetes have been shown to give 94% correct responses on average, while control subjects have an average of 67% correct responses (Eagleman et al., 2007). Therefore synaesthetes can be expected to respond correctly to at least 80% of the trials in this test.

#### Statistical procedure

For each participant, consistency score, short questionnaire score, percentage of correct responses and reaction times in the speeded consistency test were calculated. Patients were classified as synaesthetes if they (1) reported experiencing synesthesia before testing, (2) reached a consistency score <1.0 and an accuracy of >80% in the speeded consistency test. In this test, reaction times of participants classified as synaesthetes and those classified as non-synaesthetes were compared to make sure that the former did not achieve higher accuracy by taking more time for their responses. For synaesthetes reporting synesthesia for both letters and numbers, mean reaction time for all graphemes was used while for those reporting synesthesia for numbers only, mean reaction time for numbers only was used. These reaction times were compared to (1) mean reaction times for all graphemes and (2) mean reaction times for numbers only in classified non-synaesthetes using two-sample t-tests. The same comparisons were made for consistency scores and percentage of correct responses in the speeded consistency test.

The percentage of patients classified as synaesthetes in the whole group included in the study, as well as in the group of patients tested, was calculated. To exclude the possibility that the percentage of synaesthetes found in the current study might be influenced by a recruiting bias (patients with synesthesia might be more likely to participate in a study), we used the percentage of synaesthetes found in the total sample of patients included, conservatively assuming that the eight patients who could not be tested would have been classified as non-synaesthetes. A two-sided 95% Wald confidence interval was calculated for the percentage of synaesthetes in the whole sample, which was then compared with prevalence calculations of GCS in the general population. The prevalence of grapheme-color synaesthetes has been found to be 1.1% in museum visitors and 1.4% in students, when only those synaesthetes who perceived colors for both numbers and letters were counted. When those participants who perceived colors for numbers only were also counted as synaesthetes, Simner et al. found 2.0% grapheme-color synaesthetes in the university sample. As we also included both types of grapheme-color synaesthetes here, that is, those who perceive colors for letters and numbers and those perceiving colors for numbers only, we used the 2.0% as reference for our study. If GCS is more prevalent in AS patients, we would expect the 95% Wald confidence interval of our estimated percentage to range above 2.0%.

Furthermore a power calculation with nQuery7.0 based on the exact test for a single proportion was performed to investigate whether the sample size was appropriate for the comparison.

## Results

### Patients reporting GCS

Twenty-nine patients were included in the study, whereby 21 were tested. Out of those, six (two females) reported perceiving colors induced by letters and numbers or by numbers only. One of these six cases (patient 11) did not achieve a consistency score smaller than 1.0 (1.36 for all items, 1.27 for numbers). He also had only a marginal questionnaire score of 16 and described his sensations “more like feelings” than visual sensations and some numbers had “two colors at the same time” for him. It is not clear whether this is the reason for the relative inconsistency of his color choices during the test. He was therefore classified as non-synaesthete. The five remaining patients who reported perceiving synesthesia reached scores within the synaesthetic range in both the consistency test and speeded consistency test, either with regard to both letters and numbers or to numbers only (according to the type of synesthesia they reported). Out of these, one patient (patient 17) achieved a score of only 16 in the questionnaire which is just below the cut-off value of 17. He had said previous to the testing procedure that he might perceive colors for numbers and letters but that this happens more on a subconscious level. Even after completing the test he claimed that he was not sure if he really “sees” the colors, although it seemed quite clear to him which colors he should choose. He was also uncertain whether the color sensations occur automatically and whether they have always been the same, which is likely to be the reason why he responded rather conservatively to some questions in the six-point questionnaire. He further claimed to have difficulties concentrating and understanding the questions of the six-point questionnaire. As he achieved a clearly synaesthetic score of 0.75 in the consistency test and accuracy of 92.59% in the speeded consistency test (RT = 0.67 s), indicating that his color associations for graphemes are consistent as well as automatic, he was classified as a synaesthete. The four remaining patients scored clearly within the synaesthetic range in the questionnaire (21–26), as well as in the tests (see Table 1). In addition, all five patients classified as synaesthetes reported experiencing types of synesthesia other than GCS (for details see Table 2).

**Table 1:**
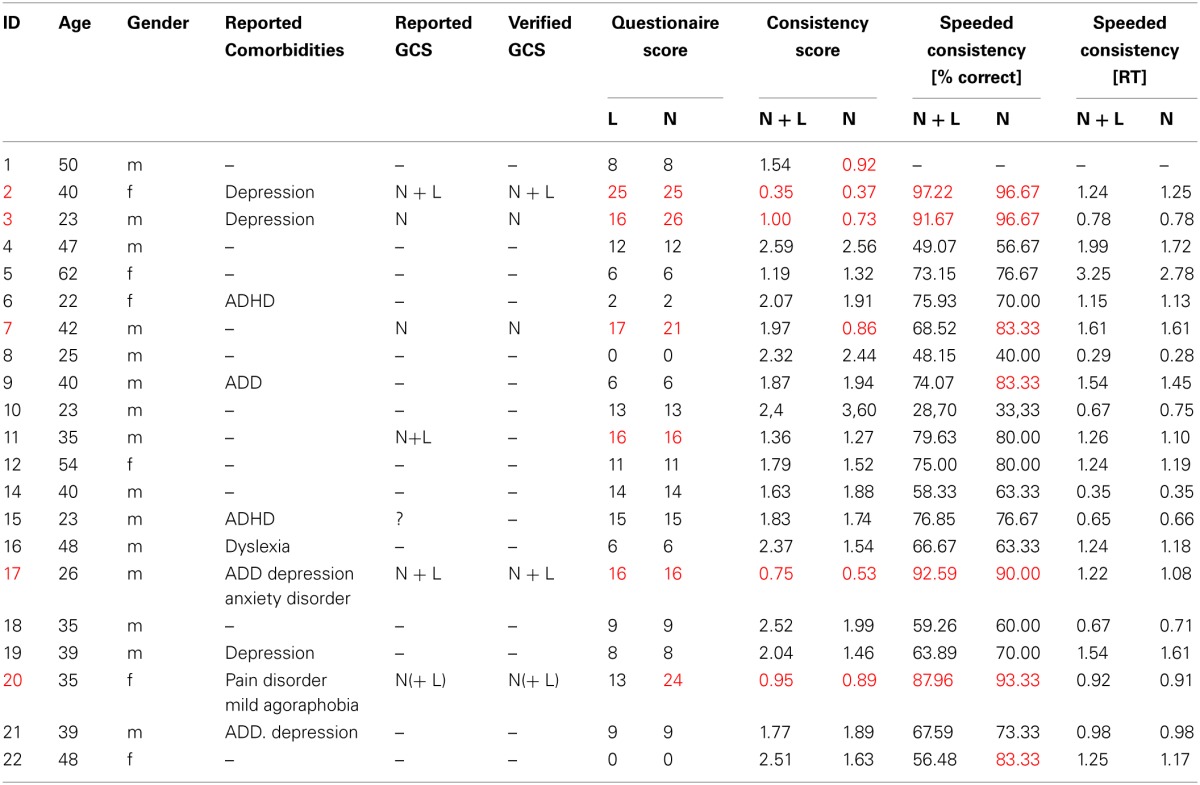
**Demographic data, questionnaire score and consistency test results of all participants**.

Prior to the tests, participants were asked whether they associate colors with numbers (N) or letters (L); they performed a consistency test, as well as a short questionnaire regarding their color associations during the test, and a speeded consistency test. In the questionnaire, synaesthetes usually have a higher score (>17), while in the consistency test lower scores indicate higher consistency which synaesthetes usually achieve (<1.0). In the speeded consistency test synaesthetes usually respond very accurately (on average 94%) compared to controls (on average 67%). Scores lying within the synaesthetic range are marked in red. Note that patient 13 was excluded from the study retrospectively, as the possibility that he had delayed speech onset could not reliably be excluded.

GCS, grapheme-color synesthesia; m, male; f, female; AD(H)D, attention deficit (Hyperactivity) disorder.

**Table 2:** **Additional synesthesia types reported by patients classified as grapheme-color synaesthetes**.

**ID**	**Age**	**Gender**	**Profession**	**Strength of GCS for numbers compared to letters**	**Additional synesthesia types reported**
2	40	f	Historian, retired	Equally strong	Word→color,
					Name→color,
					Month→color,
					Weekdays→color
3	23	m	Unemployed	Stronger for numbers	Word→shape,
					Number→pattern,
					Number→character (OLP),
					Weekdays/month→space (sequence-space synesthesia),
					Month→temperature,
					Sound→touch,
					Musical note→color,
					Musical note→number,
					Touche→motion,
					Emotion→color,
					Object→emotion,
					Voice→character,
					Color→music,
					Geometric shape→emotion
7	42	m	Journalist	Numbers only	Three-dimensional shapes→emotion,
					Colore→motion,
					Number→character (OLP),
					Other people's emotions/intentions→colored/3-dimensional shapes
17	26	m	Motor-mechanic	Equally strong	Month→color,
					Weekdays→color,
					Pain→color,
					Dates→space (sequence-space synesthesia),
					Music→color,
					Tone→color,
					Sound→color,
					Letters→character (OLP)
20	35	f	Student (sociology & media sciences)	Stronger for numbers	Numbers→gender/valence (OLP),
					Weekdays→color/emotion,
					Music→color/shape/movement/texture,
					Tone→color/movement,
					Objects→character,
					Pain→color/temperature,
					Geometrical shapes→color,
					Movement→shape,
					Weekdays/month→space (sequence-space synesthesia)

All patients classified as grapheme-color synaesthetes reported synesthesia types other than GCS, which were assessed by a questionnaire containing a list of possible inducers and concurrents.

f, female; m, male; OLP, ordinal linguistic personification.

### Patients not reporting GCS

There was one patient (patient 15) who claimed to be unsure as to whether he perceives colors induced by graphemes; he had a score of 15 in the six-point questionnaire (which is within the non-synaesthetic range, although relatively close to the cut-off value of 17), but as his test scores were clearly within the non-synaesthetic range (consistency score = 1.83, speeded consistency = 76.9% correct) he was classified as a non-synaesthete. Among the remaining patients, no others claimed to perceive colors when seeing numbers or letters and none of them reached a score higher than 17 (0–14) in the six-point questionnaire, or a score ≤1.0 in the consistency test (see Table 1, for a test example see Figure 1B).

For the 16 patients who were classified as non-synaesthetes, the consistency score was between 1.19 and 2.59 (mean = 1.99; *SD* = 0.44) and the accuracy in the speeded consistency test was between 28.70and 79.63% (mean = 63.52%; *SD* = 13.90%). The mean questionnaire score was 8.44 ± 4.99.

When looking at RGB values for numbers only, the 16 patients classified as non-synaesthetes reached consistency scores between 0.92 and 3.29 (mean = 1.73; *SD* = 0.43) and an accuracy in the speeded consistency test between 33.33 and 83.33% (mean = 67,35%; *SD* = 15.03%).

### Comparing patients classified as GC-synaesthetes with those classified as non-synaesthetes

**Figure 2:**
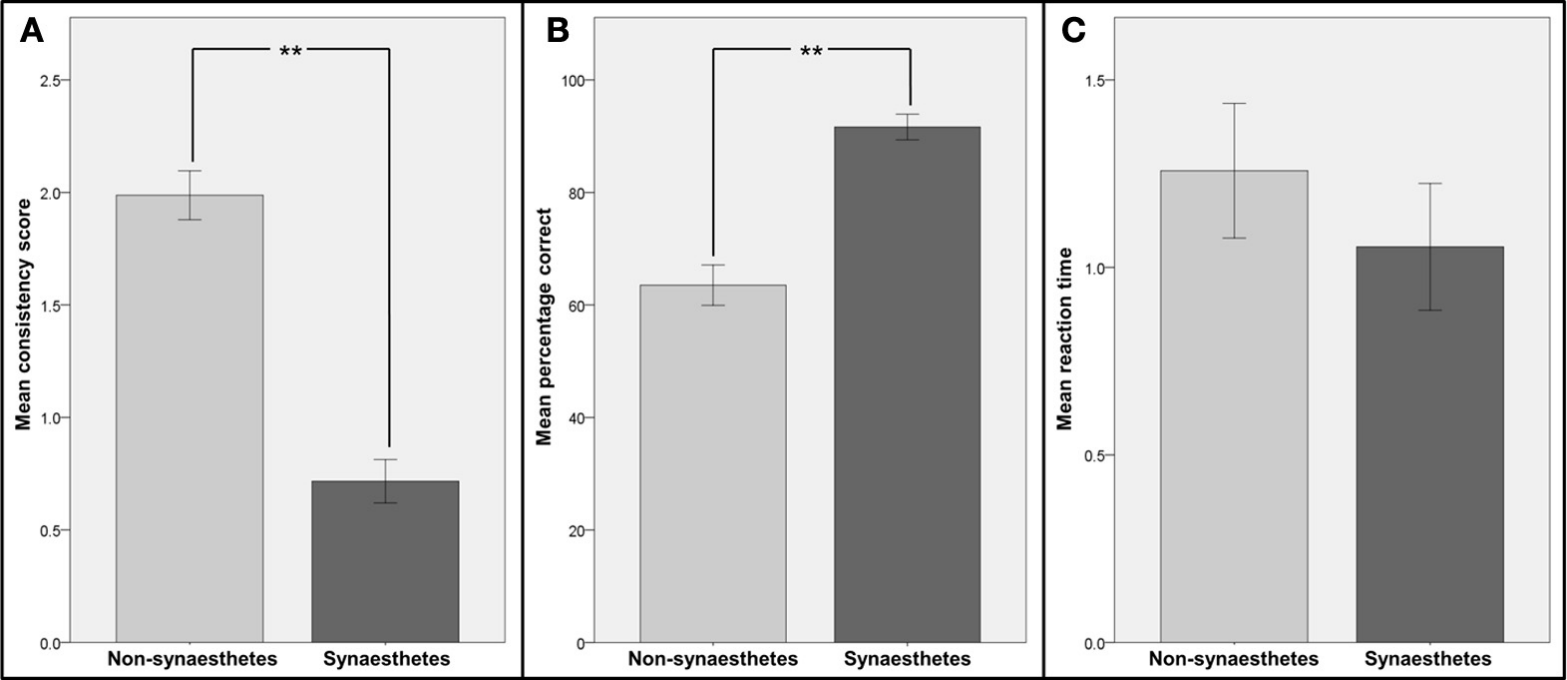
**Comparison of consistency and reaction times for patients identified as synaesthetes with those for patients identified as non-synaesthetes**. Participants were divided into two groups: those identified as synaesthetes (*N* = 5, dark gray bars) and those identified as non-synaesthetes (*N* = 16, light gray bars). **(A)** Mean consistency scores (GC-synaesthetes: mean =; SD =, non-synaesthetes: mean = 1.99; SD =), **(B)** mean percentage of correct responses in the speeded consistency test and **(C)** mean reaction times in the speeded consistency test are shown. Note that for the two synaesthetes who reported synesthesia for numbers only, the scores and reaction times for numbers only were used. Error bars represent between-subject standard errors, stars indicate level of significance.

The consistency scores, percentages of correct responses and reaction times in the speeded consistency test (both for all graphemes and numbers only) for the two groups were compared (see Figure 2). The distributions of all metrics were found to be not significantly different from normal distribution (Kolmogorov-Smirnov-Test) and therefore independent-sample t-tests were used for comparisons. Patients classified as synaesthetes were significantly more consistent in their color choices in the consistency test [*t*_(20)_ = 5.79; *p* < 0.001] and in the speeded consistency test, regardless of whether the percentages of correct responses for the synaesthetes (letters and numbers or numbers only, depending on synesthesia type) were compared to the values for all graphemes [*t*_(19)_ = 4.19; *p* < 0.001) or numbers only [*t*_(19)_ = 3.47; *p* < 0.001) in the non-synaesthete group. However the reaction times in the speeded consistency test were not significantly different between groups when comparing the synaesthetes' values to non-synaesthetes' values for all graphemes [*t*_(19)_ = 0.44; *p* = 0.67) or numbers only [*t*_(19)_ = 0.24; *p* = 0.81). There was no significant correlation between response time and accuracy (*R* = 0.23, *p* = 0.31).This strongly suggests that those patients classified as synaesthetes did not achieve higher consistency by memorizing responses.

### Statistical analysis

**Figure 3:**
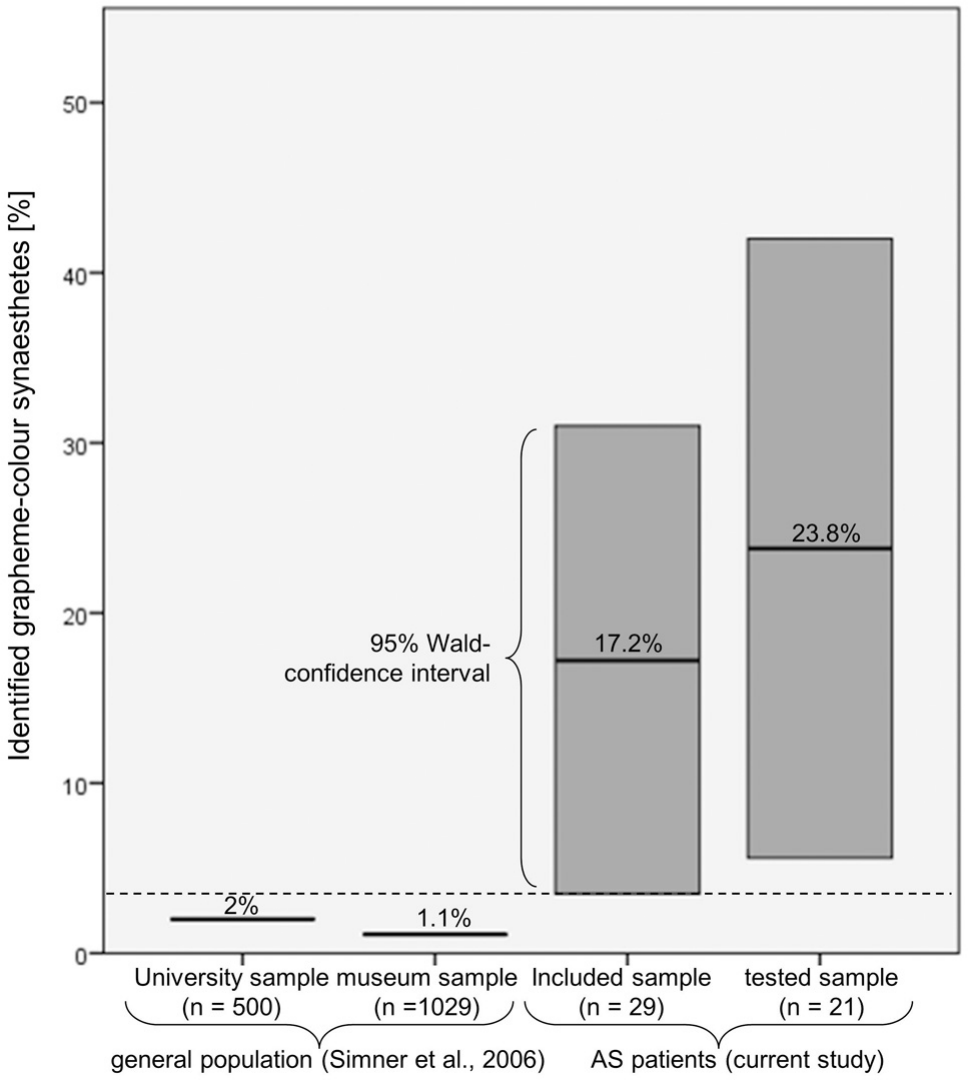
**Comparison of the percentage of patients who were identified as grapheme-color synaesthetes with the prevalence estimations in the general population**. Even when assuming that the patients who could not be tested would be classified as non-synaesthetes, the associated 95% Wald confidence interval (3.5–31.0%) still exceeds the prevalence reported by Simner et al. (2006).

All in all, six patients reported GCS and, of these, it was possible to verify five as synaesthetes: two perceiving letters and numbers equally in color, two perceiving only numbers in color and one perceiving strong colors induced by numbers and comparatively weak color associations induced by letters. These patients made up 23.8% of all patients tested and 17.2% of all patients included in the study (see Figure 3). For the more conservative estimation of 17.2%, the 95% Wald confidence interval extends from 3.5% to 31.0%, exceeding the prevalence of 1.1–2.0% found in the general population (Simner et al., 2006). Therefore the rate of synaesthetes was significantly higher in the patient group than in the general population. For the sample of tested patients the 95% Wald confidence interval extends from 5.6% to 42.0%.

The power calculation for the exact test for a single proportion with a two-sided type I error of 5% demonstrated that the sample size was large enough for the observed effect. The probability of proving a difference between 2 and 17.2% (the most conservative scenario) with 29 patients is 89%.

### Other synesthesia types

Overall, 36 types of synesthesia other than GCS were reported by patients, regardless of whether they were classified as grapheme-color synaesthetes or not (see Table 3).

**Table 3:** **List of reported synesthesia types apart from GCS**:

**Synesthesia type**	**Number of reports**	**Synesthesia type**	**Number of reports**
Sequence→space	7	Geometric shape→sound	1
Sequence→texture	1	Geometric shape→emotion	1
Day→color	4	Geometric shape→color	1
Month→color	2	Object→emotion	1
Month→temperature	1	Object→texture	1
Word→color	1	Object→charatcer	1
Word→movement	1	Electrical towers→character	1
Word→shape	2	Place→color	1
Number→pattern	1	City→color	1
Number→character (OLP)	3	Event→color	1
Sound→color	1	Touch→color	1
Sound-touch	1	Pain→temperature	2
Music→colored spapes	1	Pain→color	1
Music→odor	1	Pain→sound	1
Musical note→color	1	Emotion→color	2
Musical note→number	1	Emotion→shape	2
Color→music	2	Emotion→taste	1
Color→character	1	Movement→shape	1

With the assistance of the experimenter, all participants completed an extensive questionnaire where they were asked to report any other type of synesthesia they thought they might have by drawing lines between the inducers and concurrents listed. Patients were also encouraged to add inducers or concurrents to the list if they thought they had a type of synesthesia that was not represented. If requested, the experimenter explained any types of synesthesia which were unclear to the participant in more detail, giving some examples.

Reports from all study participants were considered here, regardless of whether they were classified as grapheme-color synaesthetes or not. Only the types of synesthesia reported are listed.

OLP, ordinal linguistic personification.

## Discussion

### Relation between synesthesia and ASC

The results of the current pilot study indicate a much higher prevalence of GCS (between 3.5 and 31%) in patients with AS than in the general population. Although the sample size in this study is too small to make assumptions about the specific prevalence of synesthesia in individuals with ASC, there is strong evidence of increased prevalence in these patients, based not only on self-report, but on verification by standardized tests. Given that co-occurrence of synesthesia and ASC by chance is *per se* very unlikely (Bor et al., 2007), the current results clearly suggest a link between AS and synesthesia. This is in line with other reports of synesthesia in ASC patients (Harrison and Hare, 2004; Bor et al., 2007; Johnson et al., 2011) and with theoretical models showing that changes in sensory perception are linked to altered neuronal connectivity in ASC (Plaisted, 2001; Mottron et al., 2006, 2013). The possible reasons for such a linkage will be discussed below, taking these models into account.

There is considerable evidence for altered sensory perception in autism. For example, “sensory hypersensitivity” (Baron-Cohen et al., 2009) has been detected in different modalities (vision, audition and touch) in ASC individuals (Bonnel et al., 2003; Bertone et al., 2005; Blakemore et al., 2006; O'Riordan and Passetti, 2006; Tommerdahl et al., 2007; Heaton et al., 2008; Simmons et al., 2009). Further, increased brain activation in primary, as well as more associative, areas connected with visual processing has been found in a wide range of different cognitive tasks, as reported by a recent meta-analysis (Samson et al., 2012). Sensory hypersensitivity in the modality of the concurrent has also been detected in synaesthetes (Banissy et al., 2009) and enhanced visual evoked potentials unrelated to synaesthetic sensation have been found in synaesthetes as well (Barnett et al., 2008a,b). Therefore both phenomena are likely to be related to altered low-level perception.

The differences in low-level perception in autism have been explained by different models. An enhanced discrimination model proposes that hyperfunctioning of low-level perception, together with a deficit in recognizing similarities between stimuli or situations on the perceptual and attentional level, causes a lack of generalization (Plaisted et al., 1998; Plaisted, 2001). Similarly, a model of enhanced perceptual functioning in autism (EPF model) suggests that the main differences in perceptual processing between autistic and non-autistic individuals are determined by more locally oriented low-level sensory perception (leading to enhanced low-level discrimination) and more autonomy of low-level processing, e.g., reduced top-down influence (Mottron et al., 2006). Furthermore, the authors propose that there is enhanced mid-level perception in more associative areas, but in a non-strategic, bottom-up or parallel fashion. This model emphasizes that variations in brain organization are the cause of the detected cognitive differences and atypical brain activation patterns toward more low-level areas (e.g., more posterior visual areas) during higher order perceptual tasks. Enhanced interaction between locally close sensory areas has not only been suggested to be a mechanism of an altered perception in autism, but also in synesthesia. Here the model of cross-activation proposes increased communication between locally adjacent brain areas involved in processing of inducer and concurrent, which would be the grapheme processing area and the color area in the fusiform gyrus in the case of GCS (Hubbard and Ramachandran, 2005).

If hyperconnectivity between sensory brain regions is the cause of altered sensory perception in autism as well as synesthesia, it is possible that a mutation in specific genes related to the development of structural or functional connections in the brain might increase the likelihood of both phenomena. For example, pruning have been suggested to be the reason for both synesthesia (Maurer, 1993; Hubbard et al., 2005) and AS (Bor et al., 2007), and increased increased (functional and structural) brain connectivity has been detected in synaesthetes (Rouw and Scholte, 2007; Jaencke et al., 2009; Haenggi et al., 2011) as well as Asperger patients (Belmonte and Yurgelun-Todd, 2003; Courchesne et al., 2005; Turner et al., 2006). Support for common genetic causes comes from a genetic marker demonstrated to be most significantly related to auditory-visual synesthesia has also been shown to been shown to be associated with ASC (Asher et al., 2009).

Besides the evidence for enhanced low-level perception and connectivity in synesthesia and ASC, there is also evidence for the involvement of associative brain regions in both phenomena. Increased activation in associative cortex regions involved in higher order sensory processing has been found in ASC individuals in the visual domain (Samson et al., 2012), while increased functional connectivity between frontal areas (Noonan et al., 2009) and between posterior cingulate and medial temporal cortex (Monk et al., 2009) has been found using fMRI. In synesthesia, the parietal cortex especially has been found to be hyperactivated in different types of synesthesia (Tang et al., 2008; Rouw et al., 2011; Neufeld et al., 2012a) and this region has also been found to be more strongly connected to the sensory areas involved in inducer- and concurrent processing (van Leeuwen et al., 2011; Sinke et al., 2012; Neufeld et al., 2012b), supporting the idea of top-down modulation of sensory areas by this higher-order associative region. It has been suggested in the so-called *two-stage model* that a combination of both increased local connectivity between sensory brain reagions and modulation of these connections by higher-order areas may be a mechanism of synesthesia (Hubbard and Ramachandran, 2005; Hubbard, 2007). The evidence for both low-level and top-down mechanisms in synesthesia implies that, while some components of synaesthetic perception might be “hard-wired,” others might be more flexible and cognitive in nature. In line with this, it has been proposed that synesthesia has genetic, as well as developmental, components; for example, the likelihood of developing synesthesia seems to be related to genetic components, whilst the specific synesthesia type and the specific inducer-concurrent inducer-concurrent pairings are developed highly individually in every synaesthete (Rouw et al., 2011).

Therefore it is likely that synesthesia does involve building associations early in life. Further, we are currently unable to distinguish between synesthesia and associations, as the latter can also occur consistently and automatically if they are overlearned rather than built spontaneously. While originally synesthesia was often described as a “merging of the senses” and the term ‘synesthesia’ itself underlines its perceptual character, researchers point out nowadays that inducers are often conceptual rather than sensory in nature (Eagleman, 2012; Juergens and Nikolić, 2012; Simner, 2012). It has been suggested recently that synesthesia might help to fill a “semantic vacuum” (which arises, for example, when a child learns letters) with meaning (Nikolić, personal communication). Evidence for this theory comes from the fact that synesthesia is much more common for abstract inducers, such as letters, numbers or days (Day, 2005; Simner, 2012), and that for these inducers the context – and therefore the semantic content – determines the synaesthetic color rather than perceptual features (Juergens and Nikolić, 2012). Therefore, synaesthetic experiences might make abstract information more concrete by acting as concrete labels and in that way make it more memorizable (Rothen et al., 2012). Synesthesia might, therefore, be one possible solution to the semantic vacuum problem, used preferentially – but not exclusively – by individuals suffering from deficits in abstract/conceptual thinking. It has been found that individuals with ASC differ from typical individuals regarding conceptual processing by being biased toward local instead of global processing, leading to a theory of ‘weak central coherence’, or a weakened drive to detect meaning by looking at the “big picture” (Happé and Frith, 2006). Difficulties in generalizing information might evoke a tendency in ASC patients to label otherwise abstract concepts with a concrete sensory experience, e.g., a color. On the other hand, enhanced memory abilities as found in individuals with ASC and Savant syndrome (Treffert, 2009) can be explained a more concrete way of processing information for these individuals. Besides the highly concrete representation of abstract concepts in savants, which has been reported anecdotally, (Murray, 2010), theoretical models showing the development of exceptional abilities related to autism point to the parallels with synesthesia (Murray, 2010; Rothen et al., 2012; Mottron et al., 2013). Specifically, the relation to synesthesia has been discussed in great detail in the context of veridical mapping, the coupling of homolog elements of recurrent isomorphic patterns, which has been proposed as an extension to the above-mentioned EPF model (Mottron et al., 2009). Veridical mapping includes, but is not restricted to, the strategic use of “if p, then q” rules (Mottron et al., 2013). The enhanced tendency to detect these rules within a system has been referred to as hypersystemizing, which has been suggested to be related to autism (Baron-Cohen et al., 2009). Interestingly, those domains which are most common as inducer categories (numbers, language, calendrical calculation, music) in synesthesia are (overlearned) linguistic sequences (Simner, 2012; Juergens and Nikolić, 2012) which are also domains that are highly “systemizable” (Baron-Cohen et al., 2009). Further, the majority of synaesthetic inducers, as well as concurrents, consist of a series of homogeneous, meaningfully ordered elements (Mottron et al., 2013). In that sense, synesthesia can be regarded as resulting from associations between corresponding members of two homologous series and, therefore, as a form of veridical mapping. If veridical mapping is enhanced in ASC, this would explain the greater tendency of those individuals to develop synesthesia.

In conclusion, shared genetic components leading to differences in brain anatomy (including local connectivity) and, in relation to that, altered cognitive mechanisms like increased veridical mapping and a greater tendency to concretize abstract information might make individuals with ASC more likely to develop synesthesia.

### Limitations of the study

The main weakness of this study is the relatively small subject number – especially compared to other prevalence investigations. However, we tried to avoid response biases (1) by including all patients diagnosed by a single institution in a certain time period and (2) by not mentioning synesthesia before commencing testing, in order to avoid response bias driven by motivation to participate (which might be enhanced in individuals who believe that they are synaesthetes). Furthermore, the aim of the current study was not to evaluate the specific prevalence of synesthesia in AS patients, but to test the hypothesis that synesthesia is more common in this group than in the general population. Our results clearly support this hypothesis. On the other hand, they do not clarify whether AS is more common among synaesthetes. The question of the prevalence of AS in synaesthetes will have to be investigated in future studies.

Another critical point of this investigation is the diagnostic procedure. There is no standard for diagnosing AS according to the DSM-IV criteria in adulthood. The “Autism Diagnostic Interview – Revised” is often used, but this interview is based purely on information from the parents, with many questions concerning the patient's childhood. However, in adults that is often difficult, as patients do not wish to involve their parents in the diagnostic process or they are not available. Also, the retrospective recall of the childhood period may be imprecise. Therefore the diagnostic process for autism in adulthood is currently problematic, especially if no diagnosis has been made in childhood. We tried to minimize this problem by a thorough exploration of the DSM-IV criteria for child- and adulthood, by interviewing and observing the patients, and by supplementing this with information from third parties or, for example, from school records.

One might argue that instead of the prevalence estimation by Simner et al. a control sample of the same sample size as our patient group could have been used as a comparison. We believe that it is appropriate to compare our data with the prevalence estimation by Simner et al., as the latter is a very reliable source due to the large sample size. In addition, we used the same instructions and questionnaires, as well as a similarly designed synesthesia test. It should be mentioned that our sample of participants differs from the sample tested by Simner et al. regarding the sex ratio: while Simner et al. tested approximately the same number of men and women in the museum study (582 female and 608 male) and more women than men in the university study (327 female and 173 male), the majority of our participants were male (16 of 21). While Simner et al. found GCS to be equally common in men and women (1.1:0.9), a previous study suggested that it is about six times more common in females (Baron-Cohen et al., 1996). If the unbalanced sex ratio in our study affected the results, it might, therefore, be expected that an even higher percentage of synaesthetes would be found in a sample with a balanced sex ratio. Interestingly, two of the five patients classified as synaesthetes were women, although only five women were tested in total.

Given that AS is believed to be far more common in boys than in girls (8:1) (Remschmidt and Kamp-Becker, 2007), one might find the male/female ratio of 3.5:1 (2.75:1 in the whole sample) in this study surprising. However, there is more recent evidence that the proportion of AS actually varies less between males and females (on average 4.6:1, but varying in different states of the U.S.A. from 2.7:1 to 7.2:1) (Centers of Disease Control and Prevention, 2012). Lying within the range found in the United States, the male/female ratio in this study is therefore quite representative.

Further work is needed to examine the relationship between AS and synesthesia by investigating a larger group of Asperger patients and the possible reasons for the linkage or similarities between the two conditions. In addition, it would also be of great interest to learn whether the prevalence of ASC, or the occurrence of autistic traits (e.g., assessed by AQ and EQ), is higher among synaesthetes.

### Conflict of interest statement

The authors declare that the research was conducted in the absence of any commercial or financial relationships that could be construed as a potential conflict of interest.
